# Living knowledge of the healing plants: Ethno-phytotherapy in the Chepang communities from the Mid-Hills of Nepal

**DOI:** 10.1186/1746-4269-4-23

**Published:** 2008-11-25

**Authors:** Arun Rijal

**Affiliations:** 1 House no.2111 "Kha", Pasanglhamu Marg, Ward no 6 Bauddha, Mahankal, Kathmandu, Nepal

## Abstract

Contribution of indigenous knowledge in developing more effective drugs with minimum or no side effects helped to realise importance of study of indigenous remedies and the conservation of biological resources. This study analysed indigenous knowledge regarding medicinal plants use among the Chepang communities from ward number 3 and 4 of Shaktikhor Village Development Committee located in the central mid hills of Nepal. Data were collected in a one-year period and included interviews with traditional healers and elders. Chepangs are rich in knowledge regarding use of different plants and were using a total 219 plant parts from 115 species including one mushroom (belonging 55 families) for medicinal uses. Out of these, 75 species had 118 different new medicinal uses and 18 of them were not reported in any previous documents from Nepal as medicinal plants. Spiritual belief, economy and limitation of alternative health facilities were cause of continuity of people's dependency on traditional healers. Change in socio-economic activities not only threatened traditional knowledge but also resource base of the area. Enforcement of local institution in management of forest resources and legitimating traditional knowledge and practices could help to preserve indigenous knowledge.

## Background

The close and traditional dependence of many indigenous and local communities on biological resources and its importance in conservation and development is now being recognised widely. The growing appreciation of the value of traditional knowledge is due to its importance to development, conservation and other wide range of uses also for other people than those who are traditionally dependent on it [1-4].

Traditional knowledge that built upon the long experiences of people was adopted in social, economic, environmental, spiritual and political practices. Since traditional knowledge is developed through a long trail and error, this could guide search for new drugs. Together with the recognition of importance of traditional knowledge, serious concern about the loss of knowledge could be observed in last few years throughout the world [4,5].

Chepangs lived a semi-nomadic life, more dependent on the forests. They have generated enormous knowledge on a large number of plants species on which they have depended for centuries. Due to this, forests were most important resources for them in terms of food, fibre, medicine, housing materials, fodder and various other needs [6,7]. The loss of knowledge could also threat the existing balance between these people and natural environment.

Despite many studies on various uses of plants a large number of plants and associated traditional uses still wait proper documentation [8]. This is perhaps because of the fact that these studies do not fully represent the wide range of environments in Nepal, where topography has created diverse ecological niches for species and stirred localized cultural constructions. In Chepang areas also documentation of ethonobotanical knowledge was limited to a few medicinal plants [9,10]. This study helped to document knowledge of several medicinal herbs use including many new reports for Nepal.

### Chepangs

There are approximately 52,000 Chepangs in 2000 [11] and their habitats are quite identical and found along the Trisuli, Narayani and Rapti Rivers and in the major catchments of their tributaries covering the adjoining frontiers of Dhading, Makwanpur, Chitwan and Gorkha districts of central Nepal [12]. Being hunter-gatherers until about 80 years ago [6], the Chepang are considered among the most primitive indigenous peoples of Nepal. They practise shifting cultivation (Slash and burn cultivation) and the evidence suggests that they are highly forest-dependent [6,10,12-14] as well as among the poorest in Nepal [12] and besides their own use of forest resources such as timber, herbs and wild food, they barter and some times sell forest products [6,12]. The forest is used as an important source of food, fibre, medicine, housing materials, fuel and fodder. They are generally considered to be shy and easily dominated by other ethnic groups [15], who have been migrating from the mountains to the lowlands for the last 40–50 years [6].

## Methodology

### Study Area

**Figure 1:**
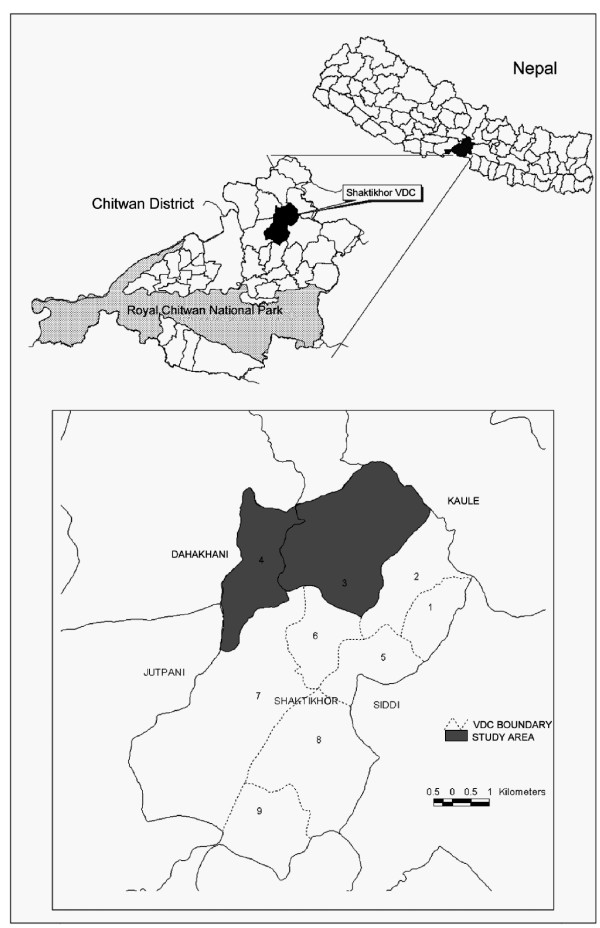
Map showing study area.

This study was conducted in two villages in ward no. 3 and 4 of Shaktikhor Village Development Committee (VDC) (27^°^40'E-27^°^48'E and 84^°^35'N-84^°^39'N) of Chitwan District in the Central Nepal (Figure 1). Though these two areas belong to the same VDC, they are in different climatic zone and have different situation of access. The population distribution is also not homogeneous but dependency on forest is common in both areas. The distribution of Chepangs is similar in these two areas. The ward number 3 is accessible from road whereas ward number 4 is on the top of the hill at one day long walking distance with no road access. The Climatic zone includes Tropical to subtropical. The altitude of this village varies from 300 m to 2500 m asl. The vegetation of this area includes lowland Sal forest, hill Sal forest, *Schima wallichi *forest, tropical riverine forest, tropical mixed broadleaved forest and subtropical mixed forest.

## Methods

Study took place between 1 June 2001 and 31 May 2002. Basically, semi-structured interviews were conducted with key informants like traditional healers for medicinal plants, and elder people and women for edible and other useful plants. Initial rapport-building visits included discussions with local leaders, traditional healers and other key informants as well as community-wide meetings introducing the research activity and its purpose. This helped to identify key informants (2 traditional healers both man and 10 elders of which 4 female and 6 male all above 60 years old). The 'artefact/interview' approach [16] was also used, i.e. asking questions about the use of plants for different purposes and making forest visits to identify the plant species. During forest visits, queries were made on plants not mentioned in the interviews, to trap the knowledge of forgotten species. Since Chepangs are very shy, a trained local assistant was used to facilitate the interviews. In interview, information on use of various medicinal plants to cure different illness, plant part used, use methods and their faith on traditional healers were acquired. Interview was mainly in Nepali but when they find difficulties to explain or understand any thing then the local assistant helped to interpret. Few old Chepangs were not confident of Nepali language so interpreter helped to interpret question as well as answer. Preparation method of any medicinal herb varies for different illness and also seriousness of the illness. Moreover, traditional faith healers were reluctant to explain mode of preparation of herbs for serious illnesses to avoid potential casualties from inexperienced person. Therefore, mode of preparation is not included in this report. Plant parts having multiple use are counted for each use. Supply column in the [see additional file 1] indicates source of supply (i.e. from wild or from the garden). Author identified plants using his more than 20 years experience from botanical studies in different parts of Nepal. Herbarium specimens were prepared following standard botanical procedure for only new specimens or specimen that needed further confirmation. The confusing species were confirmed by tallying with the herbarium at the National Herbarium and Plant Research Department (Kath), Godawari, Nepal. Plant names follows Press et al. 2000 [17]. Secondary information involves publication on information of the study area, ethnobotanical studies of Chepangs, and medicinal plants studies in Nepal. To confirm new reports for Nepal, findings were compared with all published information from Nepal including bulletins of Department of Medicinal plants of Nepal.

**Figure 2:**
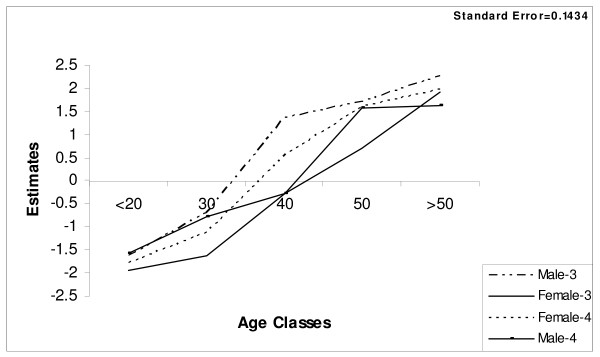
Knowledge estimates for different age and sex groups from the two wards.

A name list of permanent residents of ward no. 3 and 4 that was made available by Village Development Committee (VDC) was used to group them into sex and five age classes (< 20, 21–30, 31–40, 41–50, > 50 yrs) for each ward. From this list, 12 individuals were drawn randomly from each age class of both sex for each ward (n = 2 × 5 × 12 × 2 = 240) and interviewed for their knowledge of medicinal plant use. Total Chepang population in these two wards was 168 household i.e. 1008 individuals. The sample size represent 24% of the total Cheapang population. The knowledge difference between youth and elder people and the transmission of knowledge was analysed through mixed linear regression analysis at 95% confidence interval of knowledge for each age class, sex and ward. The use of 95% confidence interval is justified by there being a tradition that all age groups are involved in plant use and good sharing of knowledge. For several causes affecting knowledge, justifications were obtained from elder Chepangs and relevant secondary sources. Estimates from the statistical analysis of plant use knowledge for each sex of all age groups from both wards were plotted against the age groups to explain graphically the knowledge distribution among each sex from both wards for all age groups (Figure 2). Similarly, average plant use knowledge for each uses was calculated to see knowledge difference in each uses. Standard deviation of each uses for each sex was also calculated to analyse magnitude of differences within each sex of both wards (Table 1 &2).

**Table 1:** Average medicinal plant use knowledge among male and female of ward number 3 & 4 of Shaktikhor VDC.

**Ward**	**Sex**	**Medicine**
**3**	**M**	130.9 ± (64.7)
**3**	**F**	96.6 ± (63.6)
**4**	**M**	111 ± (59.1)
**4**	**F**	117.2 ± (66.6)

The values in the parenthesis are standard deviation.

**Table 2:** Models describing variation in plant use knowledge.

Model describing the variation of medicinal plant use knowledge among different age groups, sex and wards (LS Means). **N = 240, σ^2 ^= 493.81, σ = 22.22, df = 106**			
**Effect**	**Estimate**	**Standard Error**	**Pr > |t|**

Intercept	185.6	48.21	< 0.0002
Age	6.9479	0.3765	< 0.0001
Sex F	105.75	2.7610	0.0001
Sex M	122.08	2.7611	0.0001
Ward 3	113.66	2.7615	0.0001
Ward 4	114.17	2.7606	0.0001
Sex F*Ward 3	-37.55	9.259	0.0001
Sex M*Ward 3	0	.	.
Sex F*Ward 4	0	.	.
Sex M*Ward 4	0	.	.

## Results

### Plant Use

A total 219 plant parts from 115 species (belonging 55 families) including one mushroom were in use among Chepang for medicinal uses. Of them 29 are trees, 36 shrubs, 25 herbs, 21 climbers, 3 ferns and one fungi (mushroom). These belong to 105 genera and 55 families. Of the plants in use among Chepangs, 75 species had 118 different new medicinal uses for Nepal and 18 of them were not reported in any previous documents from Nepal as medicinal plants. Number of plant used to treat fever was highest (17 species) followed by wound, diarrhoea and indigestion (14 to each). Of these 115 species, 107 are wild, 7 cultivated and 1 both wild as well as cultivated. Responding question regarding faith on traditional healers, all respondent (except few youth) expressed their faith.

Plant parts use indicated that the root/rhizome had the highest use (45) followed by bark (39), fruit (32) and leaf (32) (Table 3). In some plants more than one part are used to cure various illnesses. Of these, 7 species were found cultivated, 107 species were collected from the wild and 1 species was found both in wild as well as planted. Detail results with species name, local name, part(s) used, ailments treated are tabulated [see additional file 1]. To maintain regeneration, traditional harvest practice was found leaving some flowers for seed formation, whole aerial part collection of annuals only after dispersal of seed and leaving some roots or tubers or bulbs for regeneration.

**Table 3:** Plant parts use.

**Plant part**	**No. of uses**
Bark	38
Root/rhizome	45
Tuber/bulb	10
Flower/inflorescence	2
Fruit	32
Seed/Grain	9
Oil/Butter	1
Latex/sap	11
Leaf	33
Stem/Stem fibre	17
Tender shoot	20
Whole plant	1

**Total parts used**	**219**

Of the 115 medicinal use species, 9 belong to different IUCN threat categories (Table 4). These falls within four threat categories and altogether 13 different parts of these species were in use among Chepang communities. Of the 9 threatened species, 4 are tree, 2 shrub, 2 climber and 1 herb.

**Table 4:** Threatened species in use.

**Species**	**Habit**	**IUCN Threat category**	**Plant Part Used**	**Medicinal uses**	Other uses*	**Total number of uses**
*Acacia catechu *(L.f.) Willd.	Tree	CT	Stem, bark	2	2	4
*Alstonia scholaris *(L.) R.Br.	Tree	R	Latex, stem	1	1	2
*Bergenia ciliata *(Haw.) Sternb.	Herb	CT	Rhizome	4	1	5
*Crateva unilocularis *Buch.-Ham.	Tree	R	Bark, shoot	1	2	3
*Dioscorea deltoidea *Wall. Ex Griseb	Climber	CT	Tuber	1	2	3
*Dioscorea prazeri *Prain & Burkill	Climber	CT	Tuber	1	2	3
*Oroxylum indicum *(L.) Kurz.	Tree	V	Seed	1	0	1
*Rauvolfia serpentina *(L.) Benth. ex Kurz	Shrub	E	Root, leaf	2	1	3
*Swertia chirayita *(Roxb.ex Fleming) H. Karst.	Shrub	V	Whole plant	1	1	2

*Other uses include uses other than medicinal (source: Rijal, 2007).

CT = Commercially Threatened, R = Rare, V = Vulnerable, E = Endangered.

Species use and plant part use indicated that there were many species for common illnesses like Indigestion (18 species), Fever (17 species) and wound (15 species) (Table 5).

**Table 5:** Number of plants for each illness.

**Illness**	**Plant parts in used**	**Number of plant used**
Abdominal spasm	9	9
Abortion	1	1
Antihelmintic	13	13
Asthama	2	2
Body ache	2	2
Boils	1	1
Burn	4	4
Chest pain	3	3
Cholera	2	2
Cold	8	8
Constipation	3	3
Cough	11	11
Cuts	2	2
Dealcoholisation	1	1
Diarrhoea	15	12
Dysentery	13	13
Enuresis	1	1
Fever	17	17
Fracture	7	6
Gastric	1	1
Heart pain	1	1
Heat sickness	11	11
Herpes zoster	1	1
Indigestion	18	18
Inflamation	1	1
Insecticide	4	4
Internal heat sickness	1	1
Liver problem	1	1
Loss of weight	3	3
Malaria	1	1
Malnourishment	2	2
Menopause	1	1
Miscariage	1	1
Oedema	1	1
Pneumonia	2	2
Psynocytis	3	3
Snake bite	5	5
Sprain	2	2
Scabies	1	1
Scorpion sting	2	2
Stomachic	6	6
Taenia pedis	2	2
Teeth infection	4	4
Throat problem	1	1
Tonic	6	6
Typhoid	3	3
Urine infection	2	2
Vomiting	2	2
Wound	15	15

**Total**	**219**	215*

*Since several species have multiple uses, total count counted species more than once (for each remedy) and due to that total became 215 which in reality is only 115 species.

### Knowledge distribution

Model of all ages of both sexes from two wards:

Y_i _= μ_0_+α.age_j_+β.sex_k_+γ.sex_k_(ward_l_)+ε_i_

Where i = 1......240; j = 1......54, k = 1, 2, l = 1, 2, Y = medicinal plant use knowledge (number); μ_0_, α_age(j)_, β_sex(k) _and γ_sexl(wardk) _are model parameters and ε_i _is the residual term (ε_i_~N (0,σ^2^)).

Statistical analysis model indicated that age, sex and interaction of sex and ward has significant effect on knowledge regarding medicinal plants.

Figure 2 (also Table 1 and 2) shows that men of Ward no. 3 are the comparatively most knowledgeable over the entire age spectrum, while men of Ward no. 4 are the least knowledgeable below 40 years of age. In Ward no. 4, women are generally slightly more knowledgeable than men (Table 2). In Ward no. 3, the knowledge difference between men and women increases with age whereas it decreases in Ward no. 4.

## Discussion

### Herbal Knowledge

Compare to other ethnic groups of Nepal, Chepangs of Shaktikhor were found very knowledgeable in use of plant for various need of livelihood [9,18-26]. This indicates close affinity of these people with plants of the area. The number of plant in use (115 species) was found higher compared to the reports from different ethnic groups of Nepal except the Tharu communities of Chitwan District [17]. From the use diversity aspects, it is highest (219) then earlier highest use (185) among Tharus of Chitwan [ibid.]. Although Khan, 1998 [9] also studied these communities, he was able to report only 19 medicinal plants in use but realised limitation of his study (only one day fieldwork) and suggested for more in-depth study. No new medicinal use for Nepal was reported in earlier studies of Chepangs [9,10]. Therefore, this study has contributed to bring more information on traditional medicinal plant use knowledge with the record of 120 new medicinal uses of 75 different species of which 18 species were not reported in any previous documents from Nepal as medicinal plants [9,10,19,25,27-33],].

### Dependency

Like in many other rural communities [6,7,34], their faith was reason for continuing herbal treatment. Chepangs belief that holy spirits live in plants, animals, rivers and mountains. According to their beliefs, disease and natural disasters are caused by disrespect of the spirits [7]. Due to such belief they have faith on *Pande *or *Phal *or *Janne *(traditional faith healers) and for preference to go to them to treat illness through spiritual practices. Besides, accessibility and economic reasons had also forced them to depend on traditional healing practice. The health post with only health assistant was established in this area very recently and is far from the village. The health post receives very limited medicine from the government and mainly for common diseases or wounds. Since most of the Chepangs were familiar with herbs of common remedies (fever, cough, cold, headache, stomach ache, body pain, heat sickness, constipation, internal heat sensation, weakness, indigestion, sprain, anti-helmintic, scabies, psynocytis, herpes zoster, burn, teeth infection, vomiting and dealcoholisation, minor diarrhoea/dysentery) they rather prefer to use herbs then visiting health post. In some serious cases of the illness like diarrhoea/dysentery, sprain, psynocysis, burn or vomiting, and other special cases like snake bite, scorpion sting, fracture, pneumonia, liver/heart/lung/kidney problems, typhoid, taenia pedis, malnourishment, urine or other infection, malaria, abortion, inflammation, miscarriage and cholera they visit traditional faith healer *(Pande/Pha//Janne)*. The plant parts and species use (Table 5) also indicated high number of species and plant parts use for remedy of common illnesses. They visit health posts only if the *Pande/Pha//Janne *advice in serious cases but they usually don't get much help as health post doesn't have doctor but only health assistant. As mentioned by Gurung, 1995 [7], villagers of this area also informed that there had been several cases in which people returned to the traditional healers when illness was not cured by modern medicine. The limited medical services in health post, poor economy and belief in faith on traditional healers are the cause of returning to traditional healers. Moreover, traditional practice was cheaper as payment could be made with goods like chicken or/and food grains which are available at their home.

### Conservation and plant use

The plant part use examination showed that root/rhizome is used in a large number of species followed by bark use. Root/rhizome and stem collection if not done carefully then could threaten existence of the species [35]. Debarking or collection of sap/latex could threat the tree if practiced inappropriately. Even more sensitive is the collection of root, rhizome, tuber and bulb. It is learned from the local residents that a large number of medicinal species are collected from these areas for commercial purpose and whole plant harvest makes the largest volume followed by seed, fruit, stem and tuber. The threat is more serious to the 9 IUCN listed species, of which root/rhizome/tuber is collected of 4, followed by stem and bark both of 2 (Table 4). These threatened species besides medicinal use also have several other uses including trade [36]. It is frequently claimed that commercial utilisation of non-cultivated plant species in Nepal is unsustainable; although the scientific evidence found very weak empirical support for this [37]. The traditional practice of Chepangs paid adequate attention to avoiding destructive harvesting [7]. They used to leave some roots, tubers or bulbs for regeneration purposes but elder Chepangs said the high market demand and loss of customary rights induced uncontrolled harvest of open-access resources. Market driven problems like premature collection and unsustainable harvest induced by unhealthy competition from migrated people and increased collectors from neighbouring areas could affect resource base of this area. Unhealthy competition seems to be increased by market force and that could lead to extinction of species and when plant will be out of people's context the knowledge related to that will also disappears [38]. Therefore, to protect species and their use knowledge it is important to control reckless harvest and this is only possible by enforcement of local social control mechanism. Besides, recognition of traditional knowledge by legitimising it to secure benefits related to it will encourage indigenous people to maintain traditional practices and protect natural resources.

### Knowledge distribution

Several studies from all over the world indicate that elder people know more about plant use than younger [39-43] and this corresponds to the findings of the present study (Table 2, and Figure 2). In Chepang communities also wild medicinal plants remain part of old people's context: they continue collection and preparation for utilisation.

Difference in knowledge between young and old people would be expected whereas significant difference between two adjacent age classes indicates problem of knowledge transmission. Loss of species, change in social practices, influence of migrated culture, influence of market, influence of development activities, change in life style, and policy problems are some of the important factors that potentially affect knowledge transmission [5,44-46]. Knowledge generated by elders from historic practices [47-49] and transmitted vertically to the younger generation is very much related to the affinity between family members [50-52]. Traditionally, all members of a Chepang family used to gather around the fireplace in the morning and evening, where sharing of knowledge of various things including plant use took place. Multi-generational families provided ample opportunities for sharing indigenous knowledge [53] and changes of such social structures affected knowledge transmission [54]. Moreover, some of the species seems to be extinct locally and this might have affected knowledge in young people.

Knowledge is generated from observation and implementation, i.e. learning by doing. Women in rural societies worldwide are often primarily responsible for ensuring household food security, health and family continuity [38,55] and due to that women are expected to be more rich than men in indigenous plant use knowledge [41,56-58]. However, medicinal plant knowledge analysis indicated that medicinal plant use knowledge specifically the one used to cure illness of serious nature was limited to traditional healers only. Moreover, there is clear evidence that in the homogeneous ward, men are in general more knowledgeable than women while women were more knowledgeable than men in heterogeneous ward (Figure 2, Table 1 &2). The reason for men being more knowledgeable then women in homogeneous ward (ward no. 3) is due to big difference in knowledge about medicinal plant use (Table 1), and one of the reason for this difference could be because all shaman-healers are men and do the 'serious' medicinal plant collection [7], while women concentrate on plants used for common and minor illnesses.

Men were more knowledgeable in the homogeneous community (Ward no. 3) than in heterogeneous community (ward 4) (Table 1 &2, Figure 2) also indicates erosion of indigenous knowledge due to socio-economic influences. In Ward no. 4, Chepangs' lifestyle is socially and economically influenced by the in-migrated non-Chepangs [6,7] and Stamm et al., 2004 [59] hold that the influence of an introduced culture results in loss of knowledge. More than half of the population of Ward no. 4 is non-Chepang [6,11]. Moreover, changes in life style and socio-economic status of people are reflected in a declining use of wild plants [43] which ultimately affects the knowledge transfer [60,61]. The influence of non-Chepangs on the life style of Chepangs was observed most outspoken among the younger generation. Devaluation of traditional plant use practices by non-Chepangs has developed a prestige feeling among them, making the young Chepangs embarrassed at collecting wild plant products and follow traditional practices in general, which resulted in loss of collection skills (e.g. tuber and root digging) and use knowledge [7]. Some Chepangs have married non-Chepangs and due to that changed life style and cultural practices, and the interaction between children of the two groups has also changed the cultural and social understanding of Chepang children [7]. Moreover, like in other aboriginal populations [62] Christian missionaries made several young Chepangs stop practising traditional spiritual and cultural activities, including herbal treatment from *Pande *(healer) [7] and non-Chepangs encouraged them to use modern medicine, leading to a loss of indigenous knowledge of medicinal plants.

## Conclusion

Chepang's knowledge could be useful for research as well as development activities. To protect the knowledge of traditional plant use and the benefits derived from it, the state should acknowledge folklore and legitimise its role. Species that are in traditional herbal practice should be protected because rural communities depend on them. By supporting traditional management institutions, traditional harvest practice should be encouraged to make harvest sustainable and benefit rural communities.

## Competing interests

The author declares that they have no competing interests.

## Authors' contributions

AR carried out field research, statistical analysis of the data and write up of the manuscript.

## Supplementary Material

Additional file 1 Medicinal plants and their uses. The table lists different medicinal plants with their local names, habit, part used and medicinal uses. Click here for file
