# Supplementary material for: Living knowledge of the healing plants: Ethno-phytotherapy in the Chepang communities from the Mid-Hills of Nepal
Source: J Ethnobiol Ethnomed. 2008 Nov 25;4:23. doi: 10.1186/1746-4269-4-23 (PMC2633329; doi:10.1186/1746-4269-4-23)
Supplement: Additional file 1 — Medicinal plants and their uses. The table lists different medicinal plants with their local names, habit, part used and medicinal uses. [file 1746-4269-4-23-S1.doc]

**Additional File 1. Medicinal plants and their uses.**

| **SN** | **Species** (Voucher no.*) | **Family** | **Habit** | **Local Name** | **Plant Part** | **Use** | **Supply** |
| --- | --- | --- | --- | --- | --- | --- | --- |
| 1 | *Acacia catechu* (L.f.) Willd. | Leguminosae | Tree | Khayar | Bark  Bark | Fracture  Cough | Wild |
| 2 | *Achyranthes aspera* L. | Amaranthaceae | Shrub | Jatengu, Datel | Root | Fever | Wild |
| 3 | *Aegle marmelos* (L.)Correa. | Rutaceae | Tree | Belasi | Fruit  Fruit | Heat sickness  Indigestion | Wild |
| 4 | *Aeschynanthus parviflorus* (D.Don)Spreng. (KATH 1354) | Gesneriaceae | Herb | Thirjo | Sap | Wound◘ | Wild |
| 5 | *Ageratum conyzoides* L. (KATH 0254) | Compositae | Shrub | Gandhe | Leaf  Leaf | Fever◘  Wound | Wild |
| 6 | *Alstonia scholaris* (L.) R.Br. (KATH 0539) | Apocynaceae | Tree | Chhatwan | Latex | Menopause◘ | Wild |
| 7 | *Amaranthus viridis* L. (KATH 0268) | Amaranthaceae | Herb | Ranghya | Root  Root  Tender shoot  Tender shoot | Wound◘  Cold◘  Constipation  Internal heat sensation | Wild |
| 8 | *Ananas comosus* (L.) Merr. | Bromeliaceae | Herb | Vuikatahar | Fruit | Heat sickness | Cultivated |
| 9 | *Antidesma bunius* (L.) Spreng. (KATH 1453) | Euphorbiaceae | Shrub | Archale | Bark | Dysentery◘ | Wild |
| 10 | *Antidesma acidum* Retz. (KATH 1634) | Euphorbiaceae | Tree | Amaro | Bark | Dysentery◘ | Wild |
| 11 | *Arisaema consanguineum* Schott. | Araceae | Herb | Banku | Tuber | Anti-helmintic | Wild |
| 12 | *Artemisia indica* Willd. (KATH 0294) | Compositae | Shrub | Patek | Root  Root  Root  Leaf | Dysentery  Stomachic  Wound  Insecticide◘ | Wild |
| 13 | *Asparagus racemosus* Willd. | Liliaceae | Herb | Gaidung | Tuber | Tonic | Wild |
| 14 | *Benincasa hispida* (Thunberg) Cogn.▲ (KATH 1205) | Cucurbitaceae | Climber | Kuvindo | Fruit | Heat sickness◘ | Cultivated |
| 15 | *Bergenia ciliata* (Haw.) Sternb. (KATH 1523) | Saxifragaceae | Herb | Pakhanbed | Rhizome  Rhizome  Rhizome  Rhizome | Diarrhoea  Sprain◘  Body ache  Abdominal spasm | Wild |
| 16 | *Betula alnoides* Buch.-Ham. Ex D.Don (KATH 0747) | Betulaceae | Tree | Saur | Bark | Wound◘ | Wild |
| 17 | *Brassica napus* L. (KATH 1532) | Cruciferae | Herb | Tori | Seed | Indigestion◘ | Cultivated |
| 18 | *Brassica rapa* var trilocularis (Roxb.) Kitam. (KATH 1544) | Cruciferae | Herb | Sarsiu | Seed | Indigestion◘ | Cultivated |
| 19 | *Bridelia retusa* (L.) Spreng. (KATH 0643) | Euphorbiaceae | Tree | Rapsi | Bark | Fracture◘ | Wild |
| 20 | *Butea buteiformis* (Voigt) Grierson | Leguminosae | Shrub | Dibhar | Fruit | Anti-helmintic | Wild |
| 21 | *Callicarpa macrophylla* Vahl (KATH 0628) | Verbenaceae | Shrub | Tichangs | Root  Leaf | Fever  Indigestion◘ | Wild |
| 22 | *Cannabis sativa* L. | Cannabaceae | Shrub | Ganja | Seed  Leaf | Diarrhoea/dysentery  Cold | Wild |
| 23 | *Capsicum annuum* L. (KATH 1636) | Solanaceae | Herb | Khursya | Root  Root | Indigestion  Fever◘ | Cultivated |
| 24 | *Caryopteris bicolor* (Roxb. ex Hadrwicke) Mabberly ▲ (KATH 1443) | Verbenaceae | Shrub | Balamohani | Leaf  Bark  Bark | Cold◘  Stomachic◘  Scabies◘ | Wild |
| 25 | *Cassia fistula* L. (KATH 0675) | Leguminosae | Tree | Raj Briksha | Fruit  Fruit  Stem | Diarrhoea  Vomiting◘  Throat problem | Wild |
| 26 | *Centella asiatica* (L.)Urb. (KATH 0560) | Umbelliferae | Herb | Bhui jhar, Ghodtapre | Leaf  Leaf | Asthma◘  Heat sickness | Wild |
| 27 | *Cheilanthes anceps* C.B.Clarke (KATH 0659) | Pteridaceae | Fern | Ranisinka | Tender shoot  Tender shoot | Wound◘  Abdominal spasm | Wild |
| 28 | Cipadessa baccifera (Roth) Miq. (KATH 1646) | Meliaceae | Shrub | Asinam sai | Leaf | Wound◘ | Wild |
| 29 | *Cissampelos pareira* L. (KATH 0612) | Menispermiaceae | Climber | Toroal | Stem | Indigestion◘ | Wild |
| 30 | *Cleistocalyx operculatus* (Roxb.) Merr. & Perry (KATH 0885) | Myrtaceae | Tree | Kyamna | Leaf  Leaf | Psynocytis  Dysentery◘ | Wild |
| 31 | *Cochlianthus gracilis* Benth. | Leguminosae | Climber | Boksi Kanda | Bark | Boils | Wild |
| 32 | *Colebrookea oppositifolia* Sm. (KATH 0322) | Labiatae | Shrub | Kaichak, Noryak | Leaf  Root  Root  Root  Root  Root | Fever  Indigestion  Pneumonia◘  Typhoid◘  Psynocytis Headache◘ | Wild |
| 33 | *Combretum roxburghii* Spreng. (KATH 1132) | Combretaceae | Climber | Dars | Bark | Anti-helmintic◘ | Wild |
| 34 | *Costus speciosus* (J. Konig) Sm. (KATH 0502) | Zingiberaceae | Herb | Betlauri | Tuber | Burn◘ | Wild |
| 35 | *Crateva unilocularis* Buch.-Ham. (KATH 0774) | Capparidaceae | Shrub | Sibligan | Bark | Liver problem◘ | Wild |
| 36 | *Crotalaria albida* Heyne ex Roth | Leguminosae | Herb | Bhuling | Stem | Indigestion | Wild |
| 37 | *Cucumis sativus* L. ▲ (KATH 1206) | Cucurbitaceae | Climber | Kakro | Seed | Indigestion◘ | Cultivated |
| 38 | *Cucurbita maxima* Duch.ex Poir. ▲ (KATH 1208) | Cucurbitaceae | Climber | Farsi | Seed | Indigestion◘ | Cultivated |
| 39 | *Cynodon dactylon* (L.) Pers. (KATH 0366) | Gramineae | Herb | Dub | Tender shoot | Indigestion◘ | Wild |
| 40 | *Dendrocalamus strictus* (Roxb.) Nees▲ (KATH 1312) | Gramineae | Shrub | Bansh | Sap | Enuresis◘ | Wild |
| 41 | *Desmodium oojeinense* (Roxb.) H. Ohashi | Leguminosae | Tree | Sandan | Stem | Cuts/wound | Wild |
| 42 | *Diploknema butyracea* (Roxb.) H.J. Lam (KATH 0703) | Sapotaceae | Tree | Alasi sai | Oil  Bark  Seed cake | Herpes zoster◘  Wound◘  Insecticide◘ | Wild |
| 43 | *Desmotrichum fimbriatum* Bl. ▲ (KATH 1012) | Orchidaceae | Orchid | Jiwanti | Bulb | Fever◘ | Wild |
| 44 | *Dioscorea alata* L. (KATH 0126) | Dioscoreaceae | Climber | Pangnang | Tuber | Anti-helmintic ◘ | Wild |
| 45 | *Dioscorea bulbifera* L. | Dioscoreaceae | Climber | Pas | Tuber | Anti-helmintic | Wild |
| 46 | *Dioscorea deltoidea* Wall. ex Griseb | Dioscoreaceae | Climber | Goi | Tuber | Anti-helmintic | Wild |
| 47 | *Dioscorea prazeri* Prain & Burkill ▲ (KATH 1124) | Dioscoreaceae | Climber | Jyar | Tuber | Anti-helmintic ◘ | Wild |
| 48 | *Diplocyclos palmatus* (L.) C.Jeffrey (KATH 0880) | Cucurbitaceae | Climber | Garumi | Fruit | Tonic◘ | Wild |
| 49 | *Drymaria diandra* Blume (KATH 0753) | Caryophyllaceae | Herb | Jalma, Armale | Tender shoot  Tender shoot | Psynocytis  Anti-helmintic ◘ | Wild |
| 50 | Dryoathyrium boryanum (Willd.) Ching (KATH 0636) | Aspidiaceae | Fern | Kal Niguro | Tender shoot  Rhizome | Dysentery◘  Abdominal spasm◘ | Wild |
| 51 | *Duchesnea indica* (Andrews) Focke ▲ (KATH 1025) | Rosaceae | Herb | Vui kafal | Tender shoot  Tender shoot  Tender shoot | Fever◘  Heat sickness◘  Loss of weight◘ | Wild |
| 52 | *Eclipta prostrata* (L.) L. (KATH 0559) | Compositae | Shrub | Jire jhhar, vangerijhar | Leaf  Leaf | Indigestion◘  Fever | Wild |
| 53 | *Elephantopus scaber* L. | Compositae | Herb | Mulapate | Leaf | Wound | Wild |
| 54 | *Ensete glaucum* (Roxb.) Cheesman (KATH 2354) | Musaceae | Shrub | Ban kera | Tuber  Tuber | Heat sickness◘  Urine infection◘ | Wild |
| 55 | *Entada phaseoloides* (L.) Merr. (KATH 0750) | Leguminosae | Climber | Pangro | Fruit | Anti-helmintic ◘ | Wild |
| 56 | *Erythrina stricta* Roxb. (KATH 2423) | Leguminosae | Tree | Phaleto | Bark  Bark  Bark  Bark  Bark | Fever  Typhoid◘  Pneumonia◘  Heat sickness◘  Abdominal Indigestion◘ | Wild |
| 57 | *Euphorbia hirta* L. (KATH 0859) | Euphorbiaceae | Herb | Byauli | Sap  Sap  Sap | Wound  Cough◘  Cold◘ | Wild |
| 58 | *Ficus semicordata* Buch.-Ham. Ex Sm. (KATH 0731) | Moraceae | Tree | Koksi | Sap | Heat sickness◘ | Wild |
| 59 | *Fomitopsis pinicola* (Swartz.:Fr.) Karst. ▲ (KATH 1502) | Fomitopsidaceae | Fungi | Jali chyau | Whole plant | Snake bite◘ | Wild |
| 60 | *Girardinia diversifolia* (Link)Friis (KATH 2353) | Urticaceae | Shrub | Malemau | Root  Root | Snake bite◘  Tonic◘ | Wild |
| 61 | *Holarrhena pubescens* (Buch.-Ham.) Wall. ex G.Don | Apocynaceae | Shrub | Dutyalo | Leaf | Dysentery | Wild |
| 62 | *Jatropha curcas* L. | Euphorbiaceae | Shrub | Dhuching | Sap  Stem | Burn  Teeth infection | Wild |
| 63 | *Lagerstroemia parviflora* Roxb. | Lythraceae | Tree | Chyansi | Leaf | Fever | Wild |
| 64 | *Lindera neesiana* (Wall ex. Nees) Kurz. (KATH 1234) | Lauraceae | Tree | Siltimur | Fruit  Fruit  Fruit  Fruit | Vomiting◘  Diarrhoea  Stomachic◘  Cholera◘ | Wild |
| 65 | *Mallotus nepalensis* Muell. Arg ▲ (KATH 669) | Euphorbiaceae | Tree | Phirphire | Stem | Anti-helmintic◘ | Wild |
| 66 | *Mallotus philippensis* (Lam.) Mull. Arg. (KATH 023) | Euphorbiaceae | Tree | Dusi | Leaf  Fruit  Stem | Dysentery  Abdominal spasm◘  Diarrhoea | Wild |
| 67 | *Mangifera sylvatica* Roxb. | Anacardiaceae | Tree | Taksai | Bark | Diarrhoea | Wild |
| 68 | *Mimosa pudica* L. | Leguminosae | Shrub | Kama Muja | Root | Fever | Wild |
| 69 | *Mimosa rubicaulis* Subsp. *Himalayana* (Gamble) H. Ohashi. (KATH 0640) | Leguminosae | Shrub | Rangchu, Mairang | Root  Root  Root | Wound◘  Sprain  Abdominal spasm◘ | Wild |
| 70 | *Morinda angustifolia* Roxb. ▲ (KATH 1231) | Rubiaceae | Shrub | Havang | Leaf  Leaf  Leaf | Anti-helmintic ◘  Insecticide◘  Taenia pedis◘ | Wild |
| 71 | *Mucuna nigricans* (Lour.) Steud. ▲ (KATH 203) | Leguminosae | Climber | Baldyangra | Fruit | Tonic◘ | Wild |
| 72 | *Mussaenda macrophylla* Wall. (KATH 1542) | Rubiaceae | Shrub | Dhobini | Tender shoot  Tender shoot  Tender shoot | Cold◘  Indigestion◘  Fever | Wild |
| 73 | *Myrica esculenta* Buch.-Ham. ex D.Don (KATH 1564) | Myricaceae | Tree | Brionumg | Bark | Malnourishment◘ | Wild |
| 74 | *Nephrolepis cordifolia* (L.) K.Persl (KATH 1643) | Davallidaceae | Fern | Pani amala | Rhizome | Heat sickness◘ | Wild |
| 75 | *Nicotiana tabacum* L. | Solanaceae | Herb | Chyakhala | Leaf  Leaf | Anti-helmintic Insecticide | Wild |
| 76 | *Ocimum tenuiflorum* L. | Labiatae | Herb | Tulashi | Leaf | Cough/Cold | Cultivated |
| 77 | *Oroxylum indicum* (L.) Kurz. | Bignoniaceae | Tree | Dakin | Seed | Wound | Wild |
| 78 | *Osbeckia nutans* Wall. ex C.B. Clarke | Melastomataceae | Shrub | Angeri | Root | Stomachic | Wild |
| 79 | *Paederia foetida* L. (KATH 1733) | Rubiaceae | Climber | Bire lahara | Stem  Root | Teeth infection◘  Snake bite◘ | Wild |
| 80 | *Pennisetum polystachion* (L.) Schult. ▲ (KATH 1085) | Gramineae | Herb | Yam | Root | Stomachic◘ | Wild |
| 81 | *Phyllanthus emblica* L. (KATH 0524) | Euphorbiaceae | Tree | Tausi | Fruit  Bark  Fruit | Tonic  Teeth infection◘  Cold | Wild |
| 82 | *Pilea symmeria* Wedd. ▲ (KATH 1112) | Urticaceae | Shrub | Aailanta | Bark | Diarrhoea◘ | Wild |
| 83 | *Piper longum* L. (KATH 0874) | Piperaceae | Herb | Pipla | Fruit  Fruit  Fruit | Cough  Chest pain◘  Asthma◘ | Wild |
| 84 | *Pogostemon benghalensis* (Burm.f.) Kuntze (KATH 0284) | Labiatae | Herb | Senghas | Tender shoot  Tender shoot  Tender shoot  Tender shoot | Cough  Cold  Indigestion◘  Loss of weight◘ | Wild |
| 85 | *Premna barbata* Wall. ex Schauer (KATH 0863) | Verbenaceae | Tree | Gineri | Leaf  Leaf | Urine infection◘  Heat sickness◘ | Wild |
| 86 | *Psidium guajava* L. (KATH 051) | Myricaceae | Tree | Amba | Leaf  Bark  Fruit  Seed | Diarrhoea  Diarrhoea  Diarrhoea  Cough◘ | Wild/cultivated |
| 87 | *Pueraria phaseoloides* (Roxb.) Benth. ▲ (KATH 1323) | Leguminosae | Climber | Birali lahara | Sap | Wound◘ | Wild |
| 88 | *Rhododendron arboretum* Sm. | Ericaceae | Shrub | Takro | Flower | Dysentery | Wild |
| 89 | *Rauvolfia serpentina* (L.) Benth. ex Kurz (KATH 0014) | Apocynaceae | Shrub | Chyarangro | Root  Leaf | Malaria◘  Fever | Wild |
| 90 | *Sapium insigne* (Royle) Benth. ex Hook f. | Euphorbiaceae | Tree | Rangati | Bark | Wound | Wild |
| 91 | *Saussurea sp.* | Compositae | Herb | Hyksag | Sap  Leaf | Heart pain  Cough | Wild |
| 92 | *Schima wallichii* (DC.) Korth. (KATH 0749) | Theaceae | Tree | Chyangsi | Bark  Bark  Bark  Bark  Bark  Bark | Taenia pedis◘  Gastric problem  Burn  Snake bite◘  Anti-helmintic Malnourishment◘ | Wild |
| 93 | *Scindapsus officinalis* (Roxb.) Schott (KATH 0665) | Araceae | Climber | Yuk maisai | Root  Root | Fracture◘  Abortion◘ | Wild |
| 94 | *Scoparia dulcis* L. | Scrophulariaceae | Shrub | Man | Leaf | Cough | Wild |
| 95 | *Shorea robusta* Gaertn. | Dipterocarpaceae | Tree | Raksi | Latex  Bark | Diarrhoea  Dysentery | Wild |
| 96 | *Sida rhombifolia* L. | Malvaceae | Shrub | Mechhedam | Leaf | Wound | Wild |
| 97 | *Spatholobus parviflorus* (Roxb.) Kuntze ▲ (KATH 0172) | Leguminosae | Climber | Mokare | Bark | Diarrhoea◘ | Wild |
| 98 | *Stephania japonica* (Thunb.) Miers (KATH 0507) | Menispermiaceae | Climber | Badalpate | Root  Root | Abdominal spasm  Scorpion sting◘ | Wild |
| 99 | *Sterculia villosa* Roxb. ex Sm. ▲ (KATH 0535) | Sterculiaceae | Tree | Botasi | Bark | Constipation problem of livestock◘ | Wild |
| 100 | *Swertia angustifolia* Buch.-Ham. Ex D.Don | Gentianaceae | Shrub | Goru tite | Stem | Fever | Wild |
| 101 | *Swertia chirayita* (Roxb.ex Fleming) H. Karst. | Gentianaceae | Shrub | Chiraito | Stem | Fever | Wild |
| 102 | *Syzygium cumini* (L.) Skeels | Myrtaceae | Tree | Jamun | Fruit  Leaf | Diarrhoea  Diarrhoea | Wild |
| 103 | *Terminalia alata* Heyne ex Roth (KATH 0385) | Combretaceae | Tree | Darsing | Bark  Bark  Bark  Bark  Bark  Stem  Stem | Cuts  Diarrhoea◘  Dysentery◘  Fracture◘  Stomachic◘  Burns  Inflammation | Wild |
| 104 | *Terminalia bellirica* (Gaertn.) Roxb. (KATH 0384) | Combretaceae | Tree | Lisi | Fruit  Fruit | Cough◘  Chest pain | Wild |
| 105 | *Terminalia chebula* Retz. | Combretaceae | Tree | Tupchi | Fruit  Fruit | Cough  Chest pain | Wild |
| 106 | *Thespesia lampas* (Cav.) Dalzell & Gibson (KATH 0860) | Malvaceae | Shrub | Ban kabas | Root  Root  Root  Root  Seed  Root | Dealcoholisation◘  Indigestion  Typhoid◘  Dysentery◘  Constipation◘  Fever◘ | Wild |
| 107 | *Thysanolaena maxima* (Roxb.) Kuntze (KATH 0207) | Gramineae | Herb | Chyas | Root  Root | Fever◘  Indigestion◘ | Wild |
| 108 | *Tinospora sinensis* (Lour.) Merr. | Menispermiaceae | Climber | Gurjo | Stem  Stem | Cough problem of Livestock  Heat sickness | Wild |
| 109 | *Tribulus terrestis* L. ▲ (KATH 2001) | Zygophyllaceae | Herb | Gokhur | Root  stem  Root | Body ache◘  Abdominal spasm◘  Miscarriage◘ | Wild |
| 110 | *Urtica dioica* L. (KATH 0378) | Uritcaceae | Shrub | Nelau | Root  Root  Tender shoot  Tender shoot  Stem  Leaf | Scorpion sting◘  Snake bite◘  Cold  Tonic  Fracture  Fracture | Wild |
| 111 | *Viscum album* L. | Loranthaceae | Tree | Harchul | Bark | Fracture | Wild |
| 112 | *Woodfordia fruticosa* (L.) Kurz | Lythraceae | Shrub | Daring | Flower | Dysentery | Wild |
| 113 | *Xeromphis spinosa* (Thunb.) Keay | Rubiaceae | Shrub | Main kanda | Fruit | Abdominal spasm | Wild |
| 114 | *Zanthoxylum armatum* DC. (KATH 2024) | Rutaceae | Shrub | Timur,Umpur | Fruit  Fruit  Fruit  Fruit  Fruit | Cholera◘  Oedema◘  Indigestion  Abdominal spasm  Teeth infection | Wild |
| 115 | **Unidentified** | unidentified | Climber | Sane lahara | Stem | Loss of weight◘ | Wild |

▲New medicinal plant for Nepal.

◘New medicinal use report for Nepal.

* Vaucher numbers are provided for only new medicinal plants for Nepal.
